# Supplementary material for: Naturally ornate RNA-only complexes revealed by cryo-EM
Source: Nature. 2025 May 6;643(8073):1135–42. doi: 10.1038/s41586-025-09073-0 (PMC12286853; doi:10.1038/s41586-025-09073-0)
Supplement: Supplementary file 1 — This file contains Supplementary Text 1 and 2, Supplementary Tables 1, 2 and 4, and additional references. [file 41586_2025_9073_MOESM1_ESM.pdf]

nature portfolio

https://doi.org/10.1038/s41586-025-09073-0

Supplementary information

Naturally ornate RNA-only complexes revealed by cryo-EM

In the format provided by the authors and unedited

Supplementary information for:

Naturally ornate RNA-only complexes revealed by cryo-EM

Rachael C. Kretsch[1], Yuan Wu[2], Svetlana A. Shabalina[3], Hyunbin Lee[4], Grace Nye[5], Eugene V. Koonin[3], Alex Gao[1,4], Wah Chiu[1,5,6,7,*], Rhiju Das[1,2,4,*]

[1] Biophysics Program, Stanford University, Stanford, CA USA
[2] Howard Hughes Medical Institute, Stanford University, Stanford, CA USA
[3] Computational Biology Branch, Division of Intramural Research, National Library of Medicine, National Institutes of Health, Bethesda, MD USA
[4] Department of Biochemistry, Stanford University School of Medicine, Stanford, CA USA
[5] Division of CryoEM and Bioimaging, SSRL, SLAC National Accelerator Laboratory, Menlo Park, CA USA
[6] Department of Bioengineering and James Clark Center, Stanford University, Stanford, CA USA
[7] Department of Microbiology and Immunology, Stanford University, Stanford, CA USA

*Correspondence to Wah Chiu (wahc@stanford.edu) and Rhiju Das (rhiju@stanford.edu).

**Page 3-4**	**Supplemental Text 1:** Description of *raiA* RNA motif tertiary structure.
**Page 4**	**Supplemental Text 2:** Comparative and covariation analysis of ornate large RNAs.
**Page 5-7**	**Supplemental Table 1:** Intramolecular motifs identified.
**Page 8-9**	**Supplemental Table 2:** Intermolecular interactions, base pairs, base stacks, and motifs.
Provided as additional file	**Supplemental Table 3:** Summary of the nucleotide-nucleotide covariations identified in the OLE, ROOL, and GOLLD alignments.
**Page 10**	**Supplemental Table 4:** Sequences used in this study.
Provided as additional file	**Supplemental File 1:** The multiple sequence alignment of OLE in Stockholm format.
Provided as additional file	**Supplemental File 2:** The multiple sequence alignment of ROOL in Stockholm format.
Provided as additional file	**Supplemental File 3:** The multiple sequence alignment of GOLLD in Stockholm format.
Provided as additional file	**Supplemental Video 1:** Structure of OLE dimer. The overall topology of the OLE dimer is displayed with the regions in **Fig. 1** highlighted.
Provided as additional file	**Supplemental Video 2:** Structure of ROOL nanocage. The overall topology of the ROOL nanocage is displayed with the regions in **Fig. 2** highlighted.
Provided as additional file	**Supplemental Video 3:** Structure of GOLLD nanocage. The overall topology of the GOLLD nanocage is displayed with the regions in **Fig. 3** highlighted.
Provided as additional file	**Source Data for Extended Data Figure 7:** The Bioanalyzer tables for OLE, ROOL, GOLLD, and raiA contain the intensity for the ladder used and the sample. The band location, in nucleotide units was calculated by linearly fitting the inverse migration time of the 7 highest intensity peaks to the known length of the ladder components (25, 200, 500, 1000, 2000, 4000, and 6000 nt), as is standard. The dynamic light scattering (DLS) table show the average intensity across 10 acquisitions and 2 replicates. Each column represents the DLS trace at a given temperature: data was collected at $25^{o}C$ and temperature was increased by $10^{o}C$ until $75^{o}C$. The mass photometry table lists every event recorded for each sample. The size of the RNA was calibrated using Millenium RNA Ladder (Ambion AM7150).

Supplemental Text 1: Description of *raiA* RNA motif tertiary structure.

The structure of another family of long RNAs, the *raiA* motif, from *Clostridium acetobutylicum* ATCC 824, was determined. Under similar concentrations and conditions to the other long RNAs, the raiA motif was found to be a well-ordered monomer (**Extended Data Fig. 4a**). The secondary structure of the raiA RNA is very similar to the structure proposed by Soares et al[1] based on covariance analysis and inline probing experiments (**Extended Data Fig. 4b**). The few differences are trivial, four previously proposed base pairs were not found in our structure: U19-G185, U29-A43, U50-G178, G55-U173, and an additional four base pairs were identified not previously annotated, mostly within the region for which there was no prior data from inline probing assays: G24-U50, G101-C169, C103-G168, G120-C162. Notably, although U29-A43 was proposed to be a base-pair, both bases were sites of spontaneous scission supporting our model with no base pair.

The linkers J1a/1b, J1b/1c and J3a/3b all interact in a complex tertiary interaction rigidifying P1 and P3 (**Extended Data Figure 4c**). A44 and A46 in this interaction, are members of a Z-anchor, one of many motifs found throughout the *raiA* motif structure (**Supplemental Table 1**). Around PK2, U82-A182 and G83-C181, our structure shows an interesting interaction where the strand loops around itself to form non-canonical interaction A184-G179 which facilitates the sharp turn of the backbone necessary to form this pseudoknot (**Extended Data Fig. 4d**). The backbone of the 3′ strand of PK2 is part of an interesting pocket. The pocket contains two cryo-EM densities of high signal indicating ions (**Extended Data Fig. 4e**). These are 6 Å apart. The pocket is formed by backbone atoms as well as G84, which is observed pointing into the pocket.

There are two major secondary structure variants in the *raiA* motif family; both are consistent with our 3D structure. In ~30% of *raiA* motif sequences, P7 and P8 are not present[1]. These stems appear peripheral, wrapping around the core of the RNA. There is weak density in the cryo-EM map indicating the P8-loop may interact with the P5-loop; however this region has poor local resolution, suggesting the interaction is transient or flexible and hence does not play an important stabilizing role for the atomically ordered core. Second, the loop in J1c/3a (19-23) forms a T-loop, into which A183 intercalates (**Extended Data Fig. 4f**). ~40% of *raiA* motifs have an additional stem, P2, between P1c and P3a[1], which would not support a T-loop. We hypothesize that the T-loop interaction is replaced by an A-minor interaction between the P2 minor groove and A183 and/or A184 (>97% sequence conserved[1]). Hence, we hypothesize that these secondary structure variants would likely have similar folds to the structure shown herein.

The long stretch of nucleotides, Jpk1/6, was previously identified as having many conserved nucleotides but being unpaired according to covariation and scission experiments[1]. We observe low local resolution in this region, supporting the lack of structure and hence flexibility of this region. Interestingly, this region is looped out into solution as opposed to being tucked into the core as might be expected for regions with high sequence conservation. Hence, this sequence does not seem to play a vital role in the structure of this RNA, and the sequence may be an important recognition signal for other nucleic acid or protein partners. Alternatively, it may play an

important structural role in an alternative biologically important conformation of this molecule that may be visited at different stages of this RNA's function, which remains unknown.

Supplemental Text 2: Comparative and covariation analysis of ornate large RNAs.

To gain insight into the biological significance and divergence of protein-independent RNA quaternary ensembles, we analyzed the evolutionary conservation of their intermolecular interactions across distinct families of large RNA molecules. This included comparative and covariance analyses of the intermolecular contacts identified in this study and the structural elements in their neighborhoods. The analysis of OLE, ROOL, and GOLLD showed that, despite limited sequence identity, they all exhibit extensive folding and some interactions that are long range in sequence, as confirmed by covariation statistics (**Extended Data Fig. 8a-c** and **Supplemental Files 1-3**). Covariance analysis across the three large RNA families revealed similar patterns in the distribution of covariance scores (**Extended Data Fig. 8a-c** and **Supplemental Table 3**), with significant enrichment of covariance scores at sites close to identified intermolecular interactions. Notably, most of the consensus positions lacked significant covariation scores due to the requirement of some variability to be able to observe sufficient mutations to detect covariation. Instead, pairs with significant covariation scores ($E<0.05$) tended to be located in variable regions of hairpin stems, while their loops remained highly conserved (**Supplemental Table 3**). This pattern underscores the role of covariation in maintaining structural stability despite sequence variability.

We observed that some hairpins with variable stems maintained highly conserved loops, particularly those containing A-runs, for example, B1 interaction in OLE (P4/L4) and B6 interaction (L14/P14) in GOLLD (see **Fig. 1d, 3p** and **Extended Data Fig. 8d-e**). In these examples, the four or three highly conserved A positions (**Extended Data Fig. 8d-e**), flanked by variable stems with a number of significant covariations (**Supplemental Table 3**), are indeed involved in A-minor interactions in our cryo-EM structures (B1 in **Fig. 1d**, B6 in **Fig. 3p**, respectively).

Supplemental Table 1: Intramolecular motifs identified.

RNA	Motif type	Residues involved
raiA	Z-anchor	A43, A44, A45, A46
	Kink-turn	116-124, 159-171, 106-110
	Ribose zipper	A57, A58, C90, U91
		U85, G86, G154, A155
		A99, A100, C103, C104
	U-Turn	G20, A21, A22
		A66, A67, A68
		U98, A99, A100
		U140, A141, A142
	UA-Handle	U18, U19, A23, A186
		G177, G178, A81, C51
	T-Loop	U18, U19, G20, A21, A22, A23, A186
	Intercalated T-Loop	U18, U19, G20, A21, A22, A23, A186, A183
	GNRA-Tetraloop	U140, A141, A142, U143
	A-minor	A57, G34, C90
		A100, G168, C103
	Platform	G128, U129, A130, G154
	Loop-E-Submotif	C153, G154, A155, U129, A130, G131
		U132, G133, A134, U150, A151, G152
	Bulged-G	C153, G154, A155, G128, U129, A130, G131
		U132, G133, A134, G149, U150, A151, G152
OLE	Z-anchor	A359, A360, G361, A362
	Kink-turn	67-74, 123-127, 173-179
	Ribose zipper	C165, C166, A345, A346
	U-Turn	U44, A45, A46
		G201, A202, G203
		U245, A246, A247
	UA-Handle	U272, U273, A358, A362
	GNRA-Tetraloop	G201, A202, G203, A204
	A-minor	A57, G269, C365
		A345, C166, G135
	Platform	C99, A101, G102, A95
	Tandem-GA-sheared	G71, A72, G177, A178

RNA	Motif type	Residues involved
ROOL	Z-anchor	U209, G210, U211, G212
	Kink-turn	127-135, 165-172
	Ribose zipper	C20, C21, A163, A164
		A257, A258, C288, C289
		U61, G62, A366, A367
	U-Turn	U37, A38, A39
		G101, A102, U103
		U306, G307, G308
		G364, A365, A366
		U563, U564, G565
	UA-Handle	G24, U25, A18, C49
		C373, U374, A356, G358
		G500, U501, A510, C513
		U507, U508, A503, A228
	A-minor	A163, C21, G52
		A257, C289, G327
		A185, U244, A383
		A341, U107, A98
		A366, G62, C202
		A404, G406, C352
	Platform	G397, U398, A399, G489
		G116, U117, A118, G88
	Loop-E-Submotif	G87, G88, A89, U117, A118, C119
		G84, A85, A86, A120, A121, U122
		U488, G489, A490, U398, A399, G400
	Bulged-G	G87, G88, A89, G116, U117, A118, C119
		U488, G489, A490, G397, U398, A399, G400
	Tandem-GA Sheared	G261, A262, G284, A285
	GA-minor	A378, G379, C248, G249, C250

RNA	Motif type	Residues involved
GOLLD	Ribose zipper	U17, C18, A22, A23
		A44, A45, G76, U77
		G107, C108, A278, A279
		A218, A219, C393, U394
		C292, U293, A203, A204
	U-Turn	G33, A34, A35
		G201, G202, A203
		U251, A252, A253
		U339, G340, G341
		G664, A665, A666
		U725, G726, G727
	UA-Handle	C580, U581, A572, G743
		U754, U755, A803, A805
	A-minor	A26, G97, U77
		A219, G287, C393
		A551, G428, C475
		A665, G686, C648
		A736, C732, G722
		A785, G460, U434
		A792, C461, G433
	Platform	U791, A792, A793, G784
		G694, U695, A696, G639
		G132, U133, A134, G156
		A295, C297, A298, U323
	Loop-E-Submotif	A155, G156, A157, U133, A134, U135
		U137, G138, A139, U151, A152, G153
		C638, G639, A640, U695, A696, A697
	Bulged-G	A155, G156, A157, G132, U133, A134, U135
		C638, G639, A640, G694, U695, A696, A697

Supplemental Table 2: Intermolecular interactions, base pairs, base stacks, and motifs.

RNA	Intermolecular Interaction	Motif, pair or stack type	Residues involved
OLE	B1	Base pair: W-S cis	A:A45 B:A48
		Base pair: W-W cis	A:A47 B:A47
		Stack: Parallel	A:A35 B:A45
	B2	Base pair: W-W cis	A:G97 B:C150
		Base pair: W-W cis	A:C98 B:G149
		Base pair: W-W cis	A:C100 B:G148
		Stack: Antiparallel	A:G97 B:A151
		Stack: Antiparallel	A:A101 B:G148
		Stack: Antiparallel	A:U147 B:A202
	B3	Base pair: W-W cis	A:G315 B:C318
		Base pair: W-W cis	A:G316 B:C317
		Base pair: W-W cis	A:C317 B:G316
		Base pair: W-W cis	A:C318 B:G315
ROOL	B1	Base pair: W-W cis	1:A38 2:U468
		Base pair: W-W cis	1:A39 2:U467
		Base pair: W-W cis	1:U40 2:A466
		Base pair: W-W cis	1:C41 2:G465
		Stack: Antiparallel	1:A38 2:G485
		Stack: Antiparallel	1:C41 2:A466
	B2	Base pair: S-W cis	1:G62 2:A366
	B3	Base pair: W-W cis	1:C263 2:G272
	B4	Base pair: W-S trans	1:A71 2:C334
		Base pair: W-W cis	1:G307 2:C335
		Base pair: W-W cis	1:G308 2:C334
		Base pair: W-W cis	1:U309 2:A333
		Base pair: W-W cis	1:A310 2:U332
		Stack: Antiparallel	1:G307 2:A336
		Stack: Antiparallel	1:G308 2:C335
		Stack: Antiparallel	1:A310 2:A333
		A-minor	1:A71, 2:C334, 1:G308
	B5	Stack: Antiparallel	1:A505 1':A505
	B6	Base pair: W-W cis	1:G565 2':C566
		Stack: Antiparallel	1:U564 2':A568
		Stack: Antiparallel	1:C566 2':C566

RNA	Intermolecular Interaction	Motif, pair or stack type	Residues involved
GOLLD	B1	Base pair: S-W cis	1:A589 2:A34
		Base pair: S-W trans	1:G604 2:A35
		A-minor	2:A34, 1:A589, 1:U603
		A-minor	2:A35, 1:G604, 1:U588
	B2	Base pair: W-W cis	1:G140 2:C146
		Base pair: S-H cis	1:U150 2:C146
		Stack: Antiparallel	1:G141 2:C146
	B3	Base pair: S-W cis	1:U293 2:A203
		Base pair: S-W trans	1:G388 2:A204
		A-minor	2:A203, 1:U293, 1:A387
	B4	Base pair: W-S cis	1:A409 2:U251
		Base pair: S-W cis	1:A415 2:A252
		Base pair: W-W cis	1:U414 2:A253
		Base pair: W-W cis	1:A413 2:U254
		Base pair: W-W cis	1:G412 2:C255
		Base pair: W-W cis	1:G411 2:C256
		Base pair: W-W cis	1:G410 2:C257
		Stack: Antiparallel	1:A409 2:C250
		Stack: Antiparallel	1:A413 2:C255
		Stack: Antiparallel	1:G412 2:C256
		Stack: Antiparallel	1:G411 2:C257
		Stack: Antiparallel	1:G410 2:U258
		A-minor	2:A252, 1:A415, 1:U408
	B5	Base pair: W-W cis	1:A372 2:G340
		Base pair: W-W cis	1:C371 2:G341
		Base pair: W-W cis	1:A369 2:U342
		Stack: Antiparallel	1:A372 2:G341
	B6	Base pair: S-W cis	1:A682 2:A447
		Base pair: S-W trans	1:G670 2:A448
		A-minor	2:A447, 1:A682, 1:U669
		A-minor	2:A448, 1:G670, 1:U681
	B7	Base pair: W-W cis	1:C618 2:G546
		Base pair: W-W cis	1:U617 2:A547
		Stack: Antiparallel	1:C618 2:A547
	B8	Base pair: W-W cis	1:G656 7':C657
	B9	Base pair: W-W cis	1:G675 7':C676
		Stack: Antiparallel	1:G675 7':A677
	B10	Base pair: S-W cis	1:U647 1':A763
		Base pair: W-S trans	1:A764 1':A688
		A-minor	1:A763, 1':U647, 1':A687
		A-minor	1:A764, 1':A688, 1':U646
		GA-minor	2:A145, 2:C146, 1:G140, 1:G141, 1:G142

Supplemental Table 4: Sequences used in this study.

***Genes** (T7 promoter bolded, priming site for amplification underlined, added or mutated nucleotides highlighted)*	
raiA<br>(*Clostridium acetobutylicum ATCC 824* AE001437.1/ 2985433- *2985231* [1,2])	**TTCTAATACGACTCACTATA**GGTTAAGTTAGGTTTGTGGTTGAAAGTCGATGCCAGTCGCAGGCAAAACG<br>ATCCACGTAAGTTAAACAAAGTTTTAATGAGCATGGTGCGGCTTAGAAGTAAGTCCTGCCGCTTTAGGCG<br>AGAGTATTAGTAGTGAGAGGGTAATTCCGGGTAGCGAAACTTCCAGCAGGCGAGTGTGGGGTCAAAGACC<br>AGGTCAACTAACTTA
OLE<br>(*Clostridium acetobutylicum ATCC 824* AE001437.1/ 2182120-2181516 “env-38” [2,3]) ∗	**TTCTAATACGACTCACTATA**GGTGCAGTATTCTAGTCAGGGAAATGCTTTTTGAAGGCGGGGCTAAAAAT<br>CCGCTAAAGGGCACATCGATGAAGTTCCTGGTGCTGGCCTTAGAATGCCCAGTCTTGGGCTTGTGCTGGG<br>AGTTAAAAAAGCTGGGGCACTCGCAATGGCATGCGACAAATGACCCTACTTTTGTGGAGGCCAATTATTG<br>TATATTGAGAGAGATATTCAATATACGAAATTGGGGTAAACCTGCAATGTGGTGTAAAAGCTATGTGCAG<br>TGTAGCCTGCCTTGAGTGGTATGGGGAGAGGAGATAAACAAGTCAAAAATTTTAGGCCTAAGTTTTTGTA<br>CTATTGAACTCTGAAACCTATGTTGCAAAAGAGGCTAAGAAAGCATCTAACTGTTGAGGAAAACTCCTAG<br>ACTGTTTTGGTAAAATGAGGATTGCAGTGCGGACTTAGTGGCAATTCAGTCCTGAAAGTGGCAACACTTC<br>AGCTCGGATATTAAAGGGAAACCGCTATATGGCGACGTATAGTTATTCGTGGGGAAAGCCTACTGAACCT<br>ATGCCGTAAGATTTACTTATTTTGTTACCACATTGCC
ROOL (RUMENNODE4169944_96001/22320-21654 ”env-120” [6,7]) †	**TTCTAATACGACTCACTATA**GGAATGTTTATAGACATAGCCTTGTGTATGACTGTCTAATCAACAGTGCA<br>AGGAATTAGTTGTGCTCTCAAAAGGAGTTATGTGAAGGAAAGATTAAATGGATACATTTAATTAAGTACA<br>ATTACTACAAAATCTATAATGACTGGGTAAGTCACCCGTAAGAAGGATGAGTTAGTAGAGATATAATAAT<br>ATCTTACAGTTCAACTGATGTGCAAGTTTATAAGTAGACGTAGTGTGAAAGACTTATCGCTAACGCAATA<br>GACGTTATCTCGAAAGGATAAAAGAAACCAAGAAGATATACAAATTGGTAACTTGTATAAGCCCTTGGTA<br>ATACCAAATAAGTTAGTTCCTCATGATGTCGTGGAAACATGACTATAAGATAAGTTGAATTCGATAGTAG<br>CCCAAGAGGAATCAAATGAGTAAAACAAGTATTATAGGTAAATATTATGTTTTTCTATCTGAATGATTGT<br>GCCTTGAAAAAGGTGGGTGAGGTCTGTGGGTAAAAATTAATTCCCATCTGGTTTTGGATTTTCTTCGGAA<br>AATTGGTGCAAGAAGCGTGGAGTTGCTAACCATTGCTCAGACTTGTATCGCCAGTAGCACGATAACGATA<br>TTTATGAATGTTGTAAGGTGAATAAAAGCCTATGATTTGTGAACATTCC
GOLLD<br>(JCVI SCAF 1096627009515/1239-410 env-38 [6,7])	**TTCTAATACGACTCACTATA**GGATGGCCGCCTATGGTCGTCAAATAAACTACGAAAGTAGGATAAAGTGG<br>AATAGTAAGGTTTAGCTATTCTGCGGTTTCATCTTGTAAAGATGGAGCTTGAGTAGGCAAGTGAGATATC<br>AATTTTTCTTAGTATGTGAGGGTAACACTTTAGCAGAGGAGAAAAGTTGACTGGGTCTTTGCAGAGATTC<br>AGTTGAGTTCGGAAGAGCAATAAGAATAACTCATAGAATTCAATGCGAGAGGTAGGGAACTAATCCCTCC<br>CTATAATCGCGGTATTCAATATGATAGTTTACTTAACATCAAATTGGGGAAACCCTTTAAGATAAGATAG<br>TGTACGGGTGGTGCCGTTATTATCCTTTGATAGAGTGTACCAGCACCTATCGATGAAGTAAACTTGGAAT<br>ATGGTGGTAGGGATACCACATCGGGTAGTTAAGTATTCTGTCGTTCAAAAGGCGGCGGAGCTTGTGATAG<br>ACCGCTACCTGAAACCACTGCAAAACCAAACTTATATTTGCATTTAGGATTGCAAAATCATAAAAATCAA<br>AATAAGAAAAAGTGTCTATCAGTTTTGACCGAAAGGTGTCTACATAGTAATGAGTTGTTCATTGCCACAA<br>ACACTCTCAAGGTGAATGTGATTCTTTCGAAAGGTTTCTAACACCGCAAGTGTGAATCTGCTCGGCAGGG<br>TAGAAGAAAATAAGTAAGAAGAGAGTAGGTTAAAACTCAAAGAGTGGTTCACTTAAATAACCGACATTGG<br>TTGTTACATCTCAAAAGGATGTGGAAACAAAGGGAATAGATAATCCTTTTAAAGACAACTACAATATGGT<br>GTATTCTCAGCCT
Primers	
Forward for all	TTCTAATACGACTCACTATAGG
raiA_R	TAAGTTAGTTGACCTGGTCTTTGA
OLE_R	GGCAATGTGGTAACAAAATAAGTAAAT
ROOL_R	GGAATGTTCACAAATCATAGGCTT
GOLLD_R	AGGCTGAGAATACACCATATTGTA
Sequences For AlphaFold 3 Prediction	
OapA	MKSLGKSINWTLAIAVITLVLAAIFSIVSTFMLSGVSWAVGMGIVLLIVLVGILFDTIGVAATAADEKPF<br>HAMASERLKGAKQAVAITRNADRFANFCNDVIGDISGIVSGTAATYVVIQLALQLGYGENSGQQFALSVL<br>FTSVVAALTVGGKAIGKTLAIEHSTAIIYQVGKVFYFLEEKMNINFLPNGKKKKEKNSRK
OapC	MSYEKVSQATDLVIGTKQTLKALEQEEVLEVVIAKDAEPRVVNKVEAMASVKQIPIIYVDSMKKLGKACG<br>IDVGAATVALKK
RpsU	MAETRVRKNESIDAALRRFKRSLSKEGTLAEVRKRKHYEKPSVRRKKKSEAARKRKF

∗ Due to DNA synthesis problems, OLE P1 was trimmed to a 3 base-pair stem

```
WT P1:                    AUAUAUUCAAUGUGGU--ACCACAUUGAUGUUAU
                    (((...((((((((((--))))))))))...)))
P1 of sequence used herein:          GGU--ACCACAUUGCC
                                  (((--)))
```

† Due to DNA synthesis limitations, a base pair was mutated from U-A to G-C

References

1. Soares, L. W., King, C. G., Fernando, C. M., Roth, A. & Breaker, R. R. Genetic disruption of the bacterial *raiA* motif noncoding RNA causes defects in sporulation and aggregation. *Proc. Natl. Acad. Sci. U. S. A.* **121**, e2318008121 (2024).
2. Nölling, J. *et al.* Genome sequence and comparative analysis of the solvent-producing bacterium Clostridium acetobutylicum. *J. Bacteriol.* **183**, 4823–4838 (2001).
3. Puerta-Fernandez, E., Barrick, J. E., Roth, A. & Breaker, R. R. Identification of a large noncoding RNA in extremophilic eubacteria. *Proc. Natl. Acad. Sci. U. S. A.* **103**, 19490–19495 (2006).
4. Weinberg, Z. *et al.* Detection of 224 candidate structured RNAs by comparative analysis of specific subsets of intergenic regions. *Nucleic Acids Res.* **45**, 10811–10823 (2017).
5. Hess, M. *et al.* Metagenomic discovery of biomass-degrading genes and genomes from cow rumen. *Science* **331**, 463–467 (2011).
6. Weinberg, Z., Perreault, J., Meyer, M. M. & Breaker, R. R. Exceptional structured noncoding RNAs revealed by bacterial metagenome analysis. *Nature* **462**, 656–659 (2009).
7. Yooseph, S. *et al.* The Sorcerer II Global Ocean Sampling expedition: expanding the universe of protein families. *PLoS Biol.* **5**, e16 (2007).
