# Supplementary material for: Naturally ornate RNA-only complexes revealed by cryo-EM
Source: Nature. 2025 May 6;643(8073):1135–42. doi: 10.1038/s41586-025-09073-0 (PMC12286853; doi:10.1038/s41586-025-09073-0)
Supplement: Supplementary file 2 — Reporting Summary [file 41586_2025_9073_MOESM2_ESM.pdf]

nature portfolio

Corresponding author(s): Wah Chiu
Rhiju Das

Last updated by author(s): Apr 15, 2025

Reporting Summary

Nature Portfolio wishes to improve the reproducibility of the work that we publish. This form provides structure for consistency and transparency in reporting. For further information on Nature Portfolio policies, see our Editorial Policies and the Editorial Policy Checklist.

Statistics

For all statistical analyses, confirm that the following items are present in the figure legend, table legend, main text, or Methods section.

n/a	Confirmed	
☐	☒	The exact sample size (*n*) for each experimental group/condition, given as a discrete number and unit of measurement
☒	☐	A statement on whether measurements were taken from distinct samples or whether the same sample was measured repeatedly
☒	☐	The statistical test(s) used AND whether they are one- or two-sided<br>*Only common tests should be described solely by name; describe more complex techniques in the Methods section.*
☒	☐	A description of all covariates tested
☒	☐	A description of any assumptions or corrections, such as tests of normality and adjustment for multiple comparisons
☒	☐	A full description of the statistical parameters including central tendency (e.g. means) or other basic estimates (e.g. regression coefficient) AND variation (e.g. standard deviation) or associated estimates of uncertainty (e.g. confidence intervals)
☒	☐	For null hypothesis testing, the test statistic (e.g. *F*, *t*, *r*) with confidence intervals, effect sizes, degrees of freedom and *P* value noted<br>*Give P values as exact values whenever suitable.*
☒	☐	For Bayesian analysis, information on the choice of priors and Markov chain Monte Carlo settings
☒	☐	For hierarchical and complex designs, identification of the appropriate level for tests and full reporting of outcomes
☒	☐	Estimates of effect sizes (e.g. Cohen's *d*, Pearson's *r*), indicating how they were calculated

Our web collection on statistics for biologists contains articles on many of the points above.

Software and code

Policy information about availability of computer code

Data collection	AcquireMP version 2024-R1.1, PR.PantaControl v1.8.0, Bioanlyzer 2100 Expert B.02.11.SI824, EPU 3.5
Data analysis	DiscoverMP version 2024-R1, PR.PantaAnalysis v1.8.0, CryoSparc v4.5.3, phenix 1.21, ModelAngelo (from relion v5), Infernal 1.1.2 and 1.1.5, R-scape 1.2.3, ViennaRNA 2.7.0, Muscle version 5 with OWEN program and MEGA X, Rosetta 3.10 (2020.42), Coot 0.9.8, ChimeraX with ISOLDE and Q-score v1.8, AlphaFold3 server version, DSSR 1.9.9

For manuscripts utilizing custom algorithms or software that are central to the research but not yet described in published literature, software must be made available to editors and reviewers. We strongly encourage code deposition in a community repository (e.g. GitHub). See the Nature Portfolio guidelines for submitting code & software for further information.

Data

Policy information about availability of data

All manuscripts must include a data availability statement. This statement should provide the following information, where applicable:

- Accession codes, unique identifiers, or web links for publicly available datasets
- A description of any restrictions on data availability
- For clinical datasets or third party data, please ensure that the statement adheres to our policy

The cryo-EM micrographs and particles, cryo-EM maps, and model coordinates are made available on EMPIAR, EMDB, and PDB, respectively (raiA motif: EMPIAR-12706, EMDB-48162 and PDB-9ELY; OLE: EMPIAR-12707, EMDB-48163 and PDB-9MCW; ROOL: EMPIAR-12708, EMDB-48179 and PDB-9MDS; GOLLD:

EMPIAR-12709, EMDB-48214 and PDB-9MEE). Bioanalyzer, dynamic light scattering, and mass photometry data can be found in Source Data for Extended Data Figure 7.

Research involving human participants, their data, or biological material

Policy information about studies with human participants or human data. See also policy information about sex, gender (identity/presentation), and sexual orientation and race, ethnicity and racism.

Reporting on sex and gender	No research involving human participants, their data, or biological material
Reporting on race, ethnicity, or other socially relevant groupings	No research involving human participants, their data, or biological material
Population characteristics	No research involving human participants, their data, or biological material
Recruitment	No research involving human participants, their data, or biological material
Ethics oversight	No research involving human participants, their data, or biological material

Note that full information on the approval of the study protocol must also be provided in the manuscript.

Field-specific reporting

Please select the one below that is the best fit for your research. If you are not sure, read the appropriate sections before making your selection.

☒ Life sciences ☐ Behavioural & social sciences ☐ Ecological, evolutionary & environmental sciences

For a reference copy of the document with all sections, see nature.com/documents/nr-reporting-summary-flat.pdf

Life sciences study design

All studies must disclose on these points even when the disclosure is negative.

Sample size	Sampling particle images into half maps where the sample size is simply half the total sample: this sampling into two halves is the gold standard method of sampling in cryo-EM so it was chosen such that the resolution we reported is comparable across the field. For dynamic light scattering 10 5 second acquisitions were sampled. This was chosen as the most extensive setting that can be chosen on the Prometheus Panta. Additionally, we saw reproducible results between two replicates. For mass photometry we sampled images at 330 Hz for 60 second totaling 19800. This time was chosen because when the variance of the mass peaks was monitored over time, after this 1 minute the variance no longer decreased significantly, indicating further sampling would not improve the precision of results and thus we had sampled sufficiently.
Data exclusions	Cryo-EM particles were excluded in a standard data processing pipeline in order to obtain high resolution data. All the raw data is deposited. No other data was excluded.
Replication	In cyro-EM the standard for replication is half-map reconstruction, which means the data is flip into two and two reconstruction are created. This is done for every map and the FSC curves display the resolution up to which the data is "reproducible." Additionally to plot B-factor, a measurement of quality of the cryo-EM data, we repeat this procedure with less and less data. The dynamic light scattering data was conducted in duplicate showing reproducibility between the duplicates. The mass photometry data was examined over the time course of collections showing reproducible peaks when you compare early v late frames.
Randomization	Particles are randomly allocated to half in the half-map reconstruction. Earlier in the cryo-EM data process, for filtering out bad particles, those refinement jobs are randomly initiated and run, however we manually select the best particle set to continue on with. For our mas photometry and dynamic light scattering experiments, particles all originated from the same tubes and particle were allowed to randomly diffuse through Brownian motion into our collection area for both methods. There is potential biophysical biases in for example what particle stick to the glass for longer times and hence float into our collection area less. For dynamic light scattering, the solution was mixed before split into replicates for dynamic light scattering which is also a random process.
Blinding	Blinding was not necessary in this study as no comparative work was conducted that would cause an unblinded individual to be biased.

Reporting for specific materials, systems and methods

We require information from authors about some types of materials, experimental systems and methods used in many studies. Here, indicate whether each material, system or method listed is relevant to your study. If you are not sure if a list item applies to your research, read the appropriate section before selecting a response.

Materials & experimental systems

n/a	Involved in the study
☒	☐ Antibodies
☒	☐ Eukaryotic cell lines
☒	☐ Palaeontology and archaeology
☒	☐ Animals and other organisms
☒	☐ Clinical data
☒	☐ Dual use research of concern
☒	☐ Plants

Methods

n/a	Involved in the study
☒	☐ ChIP-seq
☒	☐ Flow cytometry
☒	☐ MRI-based neuroimaging

Plants

Seed stocks	*Report on the source of all seed stocks or other plant material used. If applicable, state the seed stock centre and catalogue number. If plant specimens were collected from the field, describe the collection location, date and sampling procedures.*
Novel plant genotypes	*Describe the methods by which all novel plant genotypes were produced. This includes those generated by transgenic approaches, gene editing, chemical/radiation-based mutagenesis and hybridization. For transgenic lines, describe the transformation method, the number of independent lines analyzed and the generation upon which experiments were performed. For gene-edited lines, describe the editor used, the endogenous sequence targeted for editing, the targeting guide RNA sequence (if applicable) and how the editor was applied.*
Authentication	*Describe any authentication procedures for each seed stock used or novel genotype generated. Describe any experiments used to assess the effect of a mutation and, where applicable, how potential secondary effects (e.g. second site T-DNA insertions, mosiacism, off-target gene editing) were examined.*
